# A novel virotherapy encoding human interleukin-7 improves *ex vivo* T lymphocyte functions in immunosuppressed patients with septic shock and critically ill COVID-19

**DOI:** 10.3389/fimmu.2022.939899

**Published:** 2022-08-15

**Authors:** Morgane Crausaz, Guillaume Monneret, Filippo Conti, Anne-Claire Lukaszewicz, Jean-Baptiste Marchand, Perrine Martin, Geneviève Inchauspé, Fabienne Venet

**Affiliations:** ^1^ Department of Infectious Diseases, Transgene SA, Lyon, France; ^2^ EA 7426 Pathophysiology of injury-induced immunosuppression (PI3), Lyon 1 University/Hospices Civils de Lyon/bioMérieux, Hôpital Edouard Herriot, Lyon, France; ^3^ Hospices Civils de Lyon, Hôpital Edouard Herriot, Laboratoire d'Immunologie, Lyon, France; ^4^ Hospices Civils de Lyon, Hôpital Edouard Herriot, Service d'anesthésie-réanimation, Lyon, France; ^5^ Department of Vectorology, Transgene SA, Illkirch-Graffenstraden, France; ^6^ Centre International de Recherche en Infectiologie (CIRI), Inserm U1111, CNRS, UMR5308, Ecole Normale Supérieure de Lyon, Université Claude Bernard-Lyon 1, Lyon, France

**Keywords:** interleukin-7, virotherapy, modified virus Ankara, T lymphocytes, sepsis, immunostimulation, COVID-19

## Abstract

A majority of patients with sepsis surviving the first days in intensive care units (ICU) enter a state of immunosuppression contributing to their worsening. A novel virotherapy based on the non-propagative Modified Virus Ankara (MVA) expressing the human interleukin-7 (hIL-7) cytokine fused to an Fc fragment, MVA-hIL-7-Fc, was developed and shown to enhance innate and adaptive immunity and confer survival advantages in murine sepsis models. Here, we assessed the capacity of hIL-7-Fc produced by the MVA-hIL-7-Fc to improve *ex vivo* T lymphocyte functions from ICU patients with sepsis. Primary hepatocytes were transduced with the MVA-hIL-7-Fc or an empty MVA, and cell supernatants containing the secreted hIL-7-Fc were harvested for *in vitro* and *ex vivo* studies. Whole blood from ICU patients [septic shock = 15, coronavirus disease 2019 (COVID-19) = 30] and healthy donors (n = 36) was collected. STAT5 phosphorylation, cytokine production, and cell proliferation were assessed upon T cell receptor (TCR) stimulation in presence of MVA-hIL-7-Fc–infected cell supernatants. Cells infected by MVA-hIL-7-Fc produced a dimeric, glycosylated, and biologically active hIL-7-Fc. Cell supernatants containing the expressed hIL-7-Fc triggered the IL-7 pathway in T lymphocytes as evidenced by the increased STAT5 phosphorylation in CD3+ cells from patients and healthy donors. The secreted hIL-7-Fc improved Interferon-γ (IFN-γ) and/or Tumor necrosis factor-α (TNF-α) productions and CD4+ and CD8+ T lymphocyte proliferation after TCR stimulation in patients with bacterial and viral sepsis. This study demonstrates the capacity of the novel MVA-hIL-7-Fc–based virotherapy to restore *ex vivo* T cells immune functions in ICU patients with sepsis and COVID-19, further supporting its clinical development.

## Introduction

Sepsis is a life-threatening organ dysfunction caused by a dysregulated host response to infection (1). In a recent study about global burden of disease, 50 million of cases were reported in 2017, leading to 11 million deaths worldwide (2). Sepsis is a major public health issue and is associated with a significant economic cost and long hospital and intensive care unit (ICU) stays (3, 4). In 2017, the World Health Organization recognized sepsis as a global health priority (5).

As patients with critically ill coronavirus disease 2019 (COVID-19) develop organ injury, coagulation, and respiratory failure after severe acute respiratory syndrome coronavirus 2 (SARS-CoV-2) infection; by definition; this disease can be classified as viral sepsis (6). Thus, the current pandemic significantly increased the burden of sepsis worldwide.

Bacterial and viral (i.e., linked to COVID-19) sepsis share similarity in the immune response to the pathogen (7–9). Following infection, an hyperinflammation phase first developed, leading to early mortality and organ dysfunctions (10, 11). A majority of patients surviving this first phase enter thereafter in a prolonged immunosuppression state, increasing susceptibility to a second infection and poor organ recovery and contributing to a delayed mortality (10–12). The injury-induced immunosuppression affects both innate and adaptive immunity. Immune dysfunctions including impaired antigen presentation, increased number of immature myeloid cells, and immune alterations affect all lymphocyte subsets (11, 13).

In particular, T lymphocytes are affected by sepsis-induced apoptosis with a decreased number of both CD4+ and CD8+ T cells (14–16). In addition to lymphopenia, patients display impaired T lymphocyte function and altered phenotype described as lymphocyte exhaustion with increased co-inhibitory molecule expression and increased proportion of regulatory T cells (11, 13, 16). Decreased proliferation capacities and reduced cytokines production *ex vivo* were observed in both patients with septic shock and critically ill COVID-19 (7, 17–19).

Immunostimulating cytokine–based therapies have recently emerged as a novel therapeutic option to specifically treat patients in the sepsis-induced immunosuppression phase. They aim at stimulating the innate or adaptive immunity to restore immune-homeostasis (12, 20). Among these therapies, IL-7–based formulations are actively developed. IL-7 is a particularly relevant cytokine as it possesses significant effects on T-cell proliferation and survival, upregulation of the anti-apoptotic Bcl-2 protein, and maintenance of T cells steady metabolism (21, 22). In addition, recombinant human IL-7 (rhIL-7) administration was shown to harbor a good safety profile in different clinical trials in oncology (23, 24), chronic infectious diseases (25–27), and, more recently, in the treatment of patients with septic shock (28). In these patients, rhIL-7 administration induced a rapid and transient decreased expression of the IL-7 receptor, CD127, an increase in T lymphocyte count and in the proliferative capacity of both CD4+ and CD8+ T cells. The rhIL-7 is currently under investigation in a clinical trial (NCT04442178) including patients with lymphopenic COVID-19, and preliminary data have demonstrated a beneficial effect on T-cell count in case studies (29, 30). However, multiple successive injections of the rhIL-7 used in these trials are required due its short circulating half-life.

We have developed a novel IL-7–based virotherapy to circumvent the need for multiple injections of the cytokine and to broaden its mechanism of action. Specifically, we have used the well-established non-propagative Modified Virus Ankara (MVA) platform (31) to deliver the IL-7 cytokine in a broad range of organs in the treated host. A recombinant MVA, the MVA-hIL-7-Fc, was engineered to encode a human IL-7 fused to the human IgG2-Fc fragment to improve the *in vivo* half-life of the encoded hIL-7. The MVA-hIL-7-Fc was extensively characterized in naïve mice and in a murine model of sepsis and shown to enhance innate and adaptive immunity as well as to confer survival advantages in two distinct models of sepsis-induced immunosuppression. Side-by-side comparison with a rhIL-7-Fc counterpart illustrated a superior capacity of the vectorized formulation to increase a large array of immune cells numbers (T lymphocytes, B and NK cells, and cells from the myeloid lineage) and functions (e.g., increased capacity to trigger T-cell differentiation and to induce cytokine-producing cells) (32).

To further support the clinical development of the MVA-hIL-7-Fc in the clinic, we assessed here the capacity of the hIL-7-Fc produced by the MVA-hIL-7-Fc to improve *ex vivo* T lymphocyte functions from immunosuppressed patients with septic shock and COVID-19.

## Materials and methods

### Clinical approval, patients, and healthy volunteers' characteristics

This exploratory study was conducted on blood samples from patients admitted to the ICU of Edouard Herriot hospital (Hospices Civils de Lyon, Lyon, France). All patients were included into two prospective observational studies: IMMUNOSEPSIS4 (for patients with septic shock) and RICO (REA-IMMUNO-COVID; for patients with COVID-19). Patients with septic shock from IMMUNOSEPSIS4 cohort were identified according to the diagnostic criteria of the Third International Consensus Definitions for Sepsis (Sepsis-3 definition) and Septic Shock (1). Septic shock diagnosis was defined on the basis of the combination of an identifiable site of infection, evidence of a systemic inflammatory response, and vasopressor therapy needed to elevate mean arterial pressure ≥65 mmHg despite adequate fluid resuscitation and lactate >2 mmol/L (18 mg/dl). Patients were excluded if under 18 years of age or presented with aplasia or pre-existent immunosuppression. The Sepsis-related Organ Failure Assessment (SOFA) score and the Simplified Acute Physiology Score (SAPS II) were used to evaluate the presence of organ dysfunctions and initial severity, respectively. Clinical and biological parameters were collected, including demographic characteristics, date and cause of ICU admission, status at day 28 after inclusion, and type of infection. This project was approved by Institutional Review Board for ethics ("Comité de Protection des Personnes Ouest II", no. RCB: 2019-A00210-57). This study is registered on clinicaltrials.gov (NCT04067674). Patients with COVID-19 over 18 years admitted in ICU with a pulmonary infection with SARS-CoV-2 confirmed by Reverse Transcription-Polymerase Chain Reaction (RT-PCR) testing were included in the RICO cohort. All patients with COVID-19 received dexamethasone (6 mg/day). This study was approved by the Institutional Review Board for ethics ("Comité de Protection des Personnes Ile de France 1", no. RCB: 2020-A01079-30) and is registered on clinicaltrials.gov (NCT04392401). Oral information and written non-opposition to inclusion in both studies were mandatory before any blood sample and were recorded in patients' clinical files. Peripheral blood of patients for both immune status analysis and functional assays was collected 3 or 4 days after ICU admission for both cohorts because the immunosuppression state of patients is established at those time points (10). The immune status of patients was analyzed by measuring the CD4+ T lymphocyte count and the human leucocyte antigen DR (HLA-DR) expression on monocytes (expressed as numbers of antibodies bound per cell AB/C) as previously described (33). Peripheral blood from 36 healthy volunteers (HVs) was provided by the blood bank of Lyon (Etablissement français du sang (EFS) de Lyon, France). According to the Etablissement français du sang(EFS) standardized procedures for blood donation and to the provisions of the article R.1243–49 and following the French public health code, a written non-opposition to the use of donated blood for research purposes was obtained from HVs. Personal data of donors were anonymized at the time of blood donation.

### Engineering of the MVA-hIL-7-Fc

The engineering of the MVA-hIL-7-Fc has been previously described (32). It is based on the highly attenuated MVA (34). Briefly, a synthetic gene coding for the fusion of the human IL-7 sequence and the human IgG2-Fc synthetized by GeneArt (Regensburg, Germany) was inserted within the MVA genome. The recombinant MVA-hIL-7-Fc and the MVATGN33.1 (empty MVA used as negative control) were produced on primary chicken embryo fibroblasts (CEFs), and viral titers in plaque-forming unit (PFU) were assessed on the continuous DF-1 cell line (American Type Culture Collection (ATCC), CRL-12203).

### Cell culture and infections

DF-1 cell line was cultured as previously described (35). Primary CEFs were prepared from chicken embryo obtained from fertilized eggs (Charles River SPAFAS, Hungary) as previously described (35). HepG2 (HB-8065, ATCC, Manassas, Virginia) and ThP1 (TIB-202, ATCC) cell lines were cultured at 37°C/5%CO_2_ in Eagle's Minimum Essential Medium (ATCC) and in RPMI 1640 medium (Gibco, Waltham, Massachusetts), respectively, and supplemented with 10% fetal bovine serum (FBS) (Gibco), 2 mM L-glutamine (Gibco), penicillin (80 U/ml)/streptomycin (80 μg/L) (Gibco). Primary human hepatocytes (HEP701LN) were purchased from Biopredic international (Saint Grégoire, France) and cultured as recommended by the manufacturer. Peripheral blood mononuclear cells (PBMCs) were purified from HV blood using SepMate (STEMCELL) and Ficoll Paque density gradient (GE Healthcare, Boston, Massachusetts). Primary monocytes were purified from PBMCs using CD14+ microbeads (Miltenyi Biotec, Bergisch Gladbach, Germany) and cultured in RPMI 1640 media as for ThP1 cell line.

HepG2 cells were infected at a multiplicity of infection (MOI) of 5 with the MVA-hIL-7-Fc or the MVATGN33.1, whereas primary human hepatocytes were infected at a MOI of 1.5 and 5. The ThP1 cell line was infected at a MOI of 3, 10, and 30, and primary human monocytes were infected at MOI of 10 and 30 with both viruses. Supernatants were harvested 24 h following infection.

### ELISA quantification of the hIL-7-Fc produced by the MVA-hIL-7-Fc

The hIL-7-Fc produced in supernatants of infected cells was quantified using the human IL-7 DuoSet ELISA Development System from R&D Systems (Minneapolis, Minnesota) according to the manufacturer's instructions. The IL-7 concentration of each sample was calculated using a standard curve established with the IL-7 standard provided by R&D Systems.

### Western blot analysis

Deglycosylation of proteins was performed using PNGase F (New England Biolabs, Ipswich, Massachusetts) according to the manufacturer's instruction and using supernatants from MVA-hIL-7-Fc–infected primary human monocytes, primary human hepatocytes, HepG2, and ThP1 cells. Treated samples were loaded on a Criterion TGX Stain Free gel 4–15% (Bio-Rad, Hercules, California) at final IL-7 quantities of 0.5, 1.9, 1.9, and 0.4 ng, respectively. Proteins were transferred to Polyvinylidene fluoride (PVDF) membrane (Bio-Rad) using Transblot Turbo system (Bio-Rad) and turbo midi high–molecular weight preset program. Antibody binding was performed overnight at +4°C using IBind Flex Western system (Invitrogen, Carlsbad, California) following the manufacturer's instruction. A primary rabbit anti-human IL-7 (Abcam, clone EPR6265, Cambridge, UK) antibody and an anti-rabbit immunoglobulin HRP-conjugated (Dako, Nowy Sącz, Poland) antibody were used to detect IL-7. Horseradish peroxidase (HRP) activity was detected using the ECL Prime Western blotting Detection reagent (Amersham GE Healthcare, Boston, Massachusetts). Chemiluminescence was recorded with a Molecular Imager ChemiDOC XRS (Bio-Rad).

### hIL-7-Fc bioactivity assessment on the PB-1 cell line

IL-7-Fc functionality was assessed as previously described using the Pre-B murine 1 (PB-1) cell line (DSMZ-German Collection of Microorganisms and cell cultures, Braunschweig, Germany) dependent of IL-7 for growth (36). Dilutions of supernatant from primary hepatocytes, primary human monocytes, HepG2, and ThP1 cells infected with the MVA-hIL-7-Fc were normalized to assess the same range of hIL-7-Fc concentration. hIL-7-Fc concentration from 0.1 to 10 ng/ml containing supernatant was assessed. In addition, similar dilutions of the corresponding MVA-N33.1 control supernatants were evaluated. Supernatants were incubated for 72 h with PB-1 cells. Cellular proliferation was measured using MTT [3-(4,5-dimethylthiazol-2-yl)-2,5-diphenyltetrazolium bromide; Sigma, Darmstadt, Germany] at Optical density (OD) of 570 nm on a Spark microplate reader (TECAN, Männedorf, Switzerland). Assays were performed in duplicates.

### Supernatants and rhIL-7 used for *ex vivo* stimulation

Supernatants from primary human hepatocytes were assessed *ex vivo* on immune cells from patients or HVs. Supernatants from primary human hepatocytes infected with the MVA-hIL-7-Fc were diluted to reach a final concentration of hIL-7-Fc of 100 ng/ml. Supernatants from uninfected cells or from cells infected with the empty MVA were evaluated at the same dilution as for the MVA-hIL-7-Fc supernatants. The rhIL-7 produced in *E.coli* and purchased from R&D Systems was used as positive control at a concentration of 100 ng/ml.

### PBMCs isolation and preparation

PBMCs were plated after isolation from fresh whole blood or were frozen for further assay. For assessment of lymphocyte proliferation, fresh PBMCs were purified using the EasySep direct human PBMCs isolation kit purchased from STEMCELL (Vancouver, Canada) and were used directly. For ELISpot assay, PBMCs were isolated by Ficoll-Paque density gradient centrifugation and were washed three times with Phosphate-buffered saline (PBS) (Gibco) supplemented with 2% FBS and frozen in 90% human AB plasma (Sigma) and 10% Dimethyl sulfoxide (DMSO) (Sigma) at a concentration between 1 and 5 million cells per tube. After thawing, PBMCs were rested 24 h at 37°C before plating. Fresh or frozen PBMCs were cultured in RPMI 1640 supplemented with 2 mM L-glutamine, penicillin (80U/ml)/streptomycin (80 μg/L), and 10% human AB plasma (Sigma).

### Cytokine production assessment by ELISpot assay

Quantification of IFN-γ– and TNF-α–producing cells was performed using a Human IFN-γ/TNF-α Double-Color ELISpot kit purchased from CTL ImmunoSpot (Shaker Heights, Ohio) following the manufacturer's instruction. PBMCs were plated at 1.25 × 10^4^ and/or 2.5 × 10^4^ cells per well and stimulated with anti-CD3 (clone HIT3a, BD Biosciences, Franklin Lakes, New Jersey) and anti-CD28 (clone CD28.2, BD Biosciences) antibodies at 1 μg/ml each. To assess activity of the hIL-7-Fc in supernatant, PBMCs were incubated either with diluted supernatants from MVA-hIL-7-Fc– or MVATGN33.1–infected or –uninfected cells or directly with the rhIL-7 in addition to anti-CD3 and anti-CD28 antibodies. Following development, images were captured and analyzed on ImmunoSpot S5 MicroAnalyzer and ImmunoSpot software. ELISpot results are represented as numbers of single–TNF-α spots or single–IFN-γ spots or double–IFN-γ–TNF-α spots per millions of PBMCs.

### Intracellular staining of cytokine

Fresh peripheral blood was collected using heparinized anticoagulant tubes. Whole blood was stimulated for 3 h using a Duractive 1 stimulation kit (Beckman Coulter, Brea, California) containing phorbol myristate acetate and ionomycin to determine T lymphocyte capacities to produce IFN-γ, TNF-α, and IL-2 according to the manufacturer's instruction. To assess hIL-7-Fc, diluted supernatants from MVA-hIL-7-Fc– or empty MVA–infected cells or –uninfected cells were added to Duractive 1 stimulating agent for 3 h. Intracellular staining of CD4+ and CD8+ T cells was performed using Duraclone IF T activation (Beckman Coulter) containing lyophilized antibodies and Perfix-nc kit (Beckman Coulter) according to manufacturer's instruction. Lyophilized antibodies from the Duraclone IF T activation kit are composed of anti-CD3 AlexaFluor 750 (clone UCHT1), anti-CD4 Pacific Blue (clone 13B8.2), anti-CD8 AlexaFluor 700 (clone B9.1), anti–IFN-γ fluorescein isothiocyanate (FITC) (clone 45.15), anti–TNF-α R phycoerythrin (PE) (clone PM2), and anti–IL-2 R PE–cyanine 7 (PE-Cy7) (clone MQ1-17H12). Cells were analyzed using CantoII flow cytometer (BD Biosciences), and data were analyzed using the FlowJo software (BD Life Sciences, Ashland, Oregon).

### Assessment of lymphocyte proliferation

To assess T lymphocyte proliferation, PBMCs (1 × 10^6^ cells/ml) were stimulated through TCR using anti–CD2-CD3-CD28 antibody–coated beads (Miltenyi Biotec; ratio of beads/cells, 1:1) or phytohemagglutinin (PHA; Thermo Scientific, Waltham, Massachusetts) at 4 μg/ml. To assess activity of hIL-7-Fc in supernatants, PBMCs of patients with septic shock and COVID-19 and HVs were incubated with diluted supernatants from MVA-hIL-7-Fc– or empty MVA–infected or –uninfected cell supernatants or directly with the rhIL-7 in addition to anti–CD2-CD3-CD28 antibody–coated beads.

After 3 days of incubation, an EdU from Click-it kit (Invitrogen) was added in culture media at 10 μM for 2 h at 37°C. PBMCs were stained with anti-human CD3 PerCP Cy5-5 (Clone UCHT1, BD Biosciences), anti-human CD4 BV510 (Clone SK3, BD Biosciences), and anti-human CD8 FITC (Clone SK1, BD Biosciences). Cells were then fixed with 50 μl of fixation buffer for 15 min at room temperature and then washed with PBS containing 1% of fetal bovine serum. Cells were permeabilized with 50 μl of Click-it saponin–based solution and incubated for 15 min at room temperature (RT). The Click-it mix including Edu buffer additive, copper, PBS, and Alexa Fluor 647 azide was prepared and added on cells for 30 min at RT. CD3+, CD4+, and CD8+ T-cell proliferation was analyzed by measuring the incorporation of EdU-AF647 into cells. Cells were analyzed on CantoII flow cytometer, and data were analyzed using the FlowJo software.

### STAT5 phosphorylation staining

Signal transducer and activator of transcription 5 (STAT5) phosphorylation after stimulation with supernatants containing hIL-7-Fc or not was assessed on whole blood of patients with septic shock and COVID-19 and HVs. Whole blood of patients was stimulated for 10 min using diluted supernatants from MVA-hIL-7-Fc– or empty MVA–infected or –uninfected cells or directly with the rhIL-7. Whole blood cells were stained with anti-human CD3 PE-Cy7 (Clone SK7, BD Biosciences) and anti-human CD45 APC-H7 (Clone 2D1, BD Biosciences). Fixation, red blood cell lysis, and permeabilization steps were performed using Perfix-Expose kit (Beckman Coulter) as recommended by the manufacturer. After permeabilization, cells were stained with anti-human pSTAT5 (Clone 47/Stat5 (pY694), BD Biosciences). Canto II flow cytometer was used to analyze cells and data were analyzed using the FlowJo software.

### Statistical analysis

Results are represented as Tukey box plots or bar plots. For Tukey box plots, the line in the middle of the box corresponds to the median value. Bottom and top of the box represent the first and the third quartiles, respectively. Lower and higher extremities of the whiskers, respectively, represent the lowest data still within 1.5 interquartile range of the lower quartile and the highest data still within 1.5 interquartile range of the upper quartile. For bar plots, the top of the bar corresponds to the median value with the interquartile range. For others, the mean ± the standard deviation (SD) are represented. For clinical characteristics analysis, the Fisher exact test was used to compare categorical variables and the Mann–Whitney test to compare continuous variables. Non-parametric Mann–Whitney unpaired tests were used to assess the difference between patients and HVs. The non-parametric Friedman test was performed followed by Dunn *post hoc* pairwise multiple comparisons to compare all conditions. All statistical analyses were performed using Prism software (GraphPad, San Diego, California). Significance was accepted at p-value < 0.05.

## Results

### MVA-hIL-7-Fc–infected cells secrete a dimeric, glycosylated, and biologically active hIL-7-Fc

Following intravenous (IV) injection of MVA in mice, Fend et al. have shown, in a biodistribution study, that MVA is detected in the lungs, the liver, and the spleen of animals and particularly in antigen-presenting cells (APCs) (37). We have previously shown that an IV injection of the MVA-hIL-7-Fc in mice provides optimal pharmacokinetic and immune activities of the expressed IL-7 (32). These data support the use of the IV route for future clinical development of the product. To mimic an IV injection of the MVA-hIL-7-Fc *in vitro*, human hepatic or monocytic cell lines (HepG2 or ThP1, classically used cell lines when studying hepatocytes or monocytes, respectively) or primary cells were infected by the MVA-hIL-7-Fc, and properties of the secreted hIL-7-Fc were analyzed.

**Figure 1:**
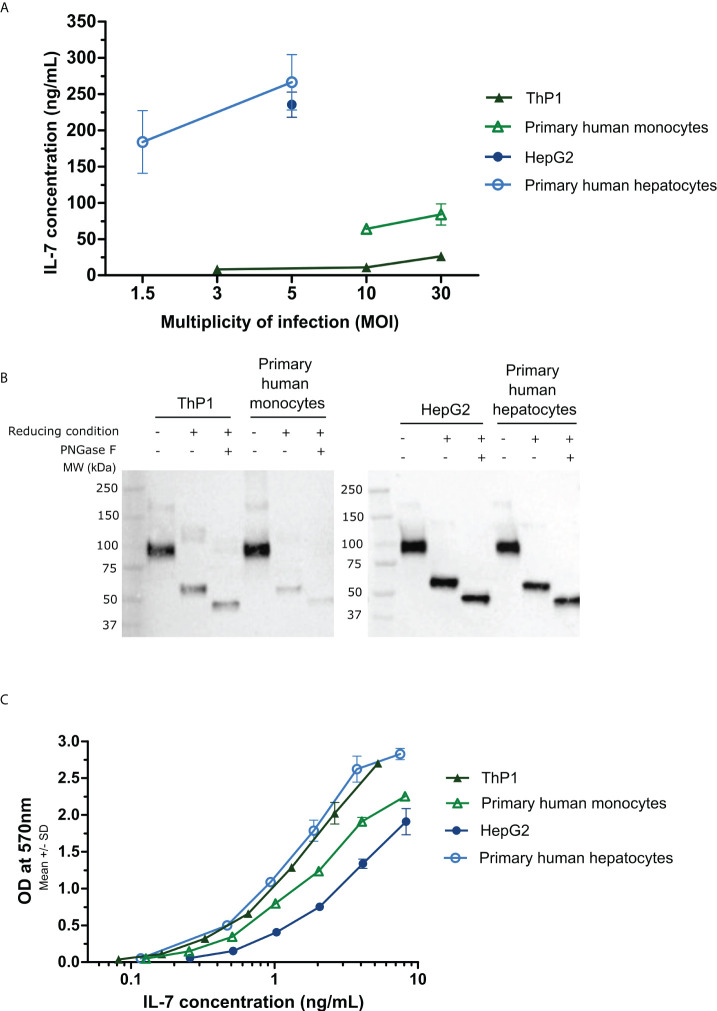
Quantification, biological activity, and Western blot analysis of the MVA-secreted hIL-7-Fc in cell supernatants following *in vitro* infection with the MVA-hIL-7-Fc. Primary human hepatocytes, HepG2 cells, primary human monocytes, and ThP1 cells were infected *in vitro* by the MVA-hIL-7-Fc (M-hIL-7-Fc) or empty MVA (M-empty) and culture supernatants harvested at 24 (h) **(A)** ELISA quantification of hIL-7-Fc. Different multiplicities of infection (MOI) of the MVAs were used depending on the cells infected. The detection range of the standard was detected between 7.18 and 500 pg/ml. **(B)** Western blot analysis of the MVA-produced hIL-7-Fc following PNGase treatment (N-glycosylation analysis) and under reducing condition (dimerization analysis). **(C)** The biological activity of hIL-7-Fc was evaluated using serial dilutions of supernatants containing hIL-7-Fc on pre-B cell line PB-1. Data are represented as means ± standard deviations of biological duplicates.

The hIL-7-Fc was detected in supernatants of all four transduced cells albeit to different levels (**Figure 1A**). The lowest quantities of hIL-7-Fc were detected in the supernatant of ThP1 cells (8.2, 10.9, and 26.3 ng/ml for MOI of 3, 10, and 30, respectively), whereas infected primary monocytes produced higher levels (64.2 and 83.9 ng/ml for MOI of 10 and 30, respectively). In a notable contrast, much higher levels of the protein were detected in hepatic cells: 235.5 ng/ml in the supernatant of HepG2 cells (analyzed only after MOI of 5) and 260 ng/ml in supernatants of primary hepatocytes (MOI of 5). No hIL-7-Fc was detected in supernatant following infection with an empty MVA in all cell types (data not shown).

To determine the glycosylation and the dimerization level of the hIL-7-Fc produced in cell supernatants after MVA-hIL-7-Fc infection, a Western blot analysis was performed (**Figure 1B**). Supernatants were treated under reducing or non-reducing conditions with or without PNGase treatment. For all supernatants, a band of approximately 45 kDa was observed under reducing condition with the PNGase treatment corresponding to the theoretical molecular weight of the monomeric deglycosylated protein (i.e., 43 kDa, predicted from the primary structure). A band of approximately 55 kDa corresponding to the monomeric glycosylated hIL-7-Fc with four expected N-glycosylation sites (three on the IL-7 and one on the Fc sequence) was detected for all cell types. Under non-reducing condition, a band of approximately 100 kDa corresponding to the glycosylated dimer (dimerization through Fc fragment) was detected.

Bioactivity assessment of the secreted cytokine was performed using the PB-1 cell line dependent of IL-7 for growth (36) (**Figure 1C**). Regardless the initial concentration, the same range of hIL-7-Fc concentration (and its empty MVA supernatant control, data not shown) was used to determine the effective dose 50 (ED_50_). Infections with the MVA-hIL-7-Fc of all cell types produced a biologically active protein. For cells from the liver origin, HepG2 cell and primary hepatocyte supernatants displayed an hIL-7-Fc with an ED_50_ of 3 and 0.91 ng/ml, respectively. Secreted hIL-7-Fc from infected ThP1 cells and primary monocytes displayed an ED_50_ of 1.0 and 1.6 ng/ml. Minor differences of activity were detected between the cell types and are in the range obtained with the commercialized rhIL-7 from *E. coli* (ED_50_ of 0.1–0.5 ng/ml) and with the counterpart rhIL-7-Fc tested in our earlier study (ED_50_ of 0.68 ng/ml) (32). Difference of activities might be explained by the lack of standardization of this cellular assay. No activity was detected when empty MVA supernatants from the different cell types were assessed.

Overall, whether produced in established cell lines or in primary cells from monocytic and hepatic origin, the MVA-expressed and -secreted hIL-7-Fc display comparable bioactivity and biochemical characteristics. In contrast, levels of produced hIL-7-Fc differ sharply, primary hepatocytes producing the highest protein concentration. To next evaluate immune activities of the MVA-secreted hIL-7-Fc on immune cells of sepsis patients, only supernatants from primary hepatocytes were tested.

### Patients with septic shock and COVID-19 display T lymphocyte dysfunctions

Fifteen patients with septic shock and 30 patients with COVID-19 in ICU were enrolled. Clinical characteristics are presented in the **Table 1**. The median age of all patients was 65 years old (Interquartile range (IQR) [54–73]). Patients with COVID-19 and septic shock presented no statistical difference regarding clinical characteristics including sex, body mass index, and comorbidities. At ICU admission, severity was more pronounced in the septic shock cohort compared with the COVID-19 cohort illustrated by significantly higher clinical scores including SAPS II and SOFA scores (for the SAPS II score, 60 [45.5–69] versus 26.5 [23.2–38.5], p < 0.001; for the SOFA score, 12 [7–14] versus 3.5 [1–8], p < 0.0001). Nevertheless, higher proportion of secondary nosocomial infections was reported in the COVID-19 group than in patients with bacterial sepsis (36.67% versus 13.3%). The 28-day mortality and ICU length of stay were greater in the COVID-19 group than in the septic shock cohort, but no statistical difference was found (for 28-day non-survivors 16.67% versus 6.7% and for the days in ICU a median of 15 versus 5).

**Table 1:** Demographic, clinical, and immunological data of patients with septic shock and COVID-19.

Parameters	All patients (n = 45)	Patients with septic shock (n = 15)	Patients with COVID-19 (n = 30)	P-value
**Age (years)**	65 [54–73]	73 [64–77]	62 [53–69]	0.012
**Gender, feminine n (%)**	21 (46.7)	7 (46.7)	14 (46.7)	1
**Body mass index (kg/m3)**	29.8 [23.8–34.0]	26.6 [23.1–35.2]	30.2 [25.3–33.9]	0.911
**Comorbidities n (%)**	27 (65.8)	10 (66.7)	17 (56.7)	0.747
**SAPS II score**	35 [24.2–55.2]	60[45.5–69]	26.5 [23.2–38.5]	<0.0001
**SOFA score**	7 [1.75–11.25]	12 [7–14]	3.5 [1–8]	0.0002
**Site of infection n (%)**				NA
Pulmonary	31 (68.9)	1 (6.7)	30 (100)	
Abdominal	5 (11.1)	5 (33.33)	0 (0)	
Others	9 (20)	9 (60)	0 (0)	
**28 days non-survivors n (%)**	6 (13.3)	1 (6.7)	5 (16.67)	0.646
**Secondary nosocomial infections n (%)**	13 (28.9)	2 (13.3)	11 (36.67)	0.164
**Days in ICU**	11 [5–30]	5 [4–16]	15 [6–32]	0.055
**Immunological parameters**				
HLA-DR on monocyte (AB/cell)	6,793 [4,789–10,239]	4,905 [3,311–7,686]	8,296 [5,445–11,027]	0.0373
Absolute CD4+ T-cell count (cells/μl)	275 [188–595]	479 [233–819]	238 [155–490]	0.057

Blood samples of 15 patients with septic shock and 30 patients with COVID-19 were analyzed in the study. Categorical data are presented as numbers and percentages. Continuous data are presented as medians and interquartile ranges [Q1–Q3]. Sequential organ failure assessment (SOFA) and simplified acute physiology score (SAPS) II were calculated during the first 24 h after ICU admission. Absolute CD4+ T-cell count and HLA-DR expression on monocytes (mHLA-DR, corresponding to anti-HLA-DR antibodies bound per monocytes AB/C) were determined on day 3. The references values of our labs are mHLA-DR> 15,000 AB/C; CD4+ cells/μl, 336–1,126. Fisher exact test was used to compare categorical variables and Mann–Whitney test to compare continuous variable.

ICU patients with septic shock and COVID-19 display characteristics of injury-induced immunosuppression at day 3 or 4 after ICU admission as shown in the **Table 1**. Most patients display a decreased HLA-DR expression on monocyte (mHLA-DR) (median of 6,793 [4,789–10,239]) and a marked CD4+ T lymphopenia (median of 275 [188–595] CD4+ cells/μl) compared with normal values (>15,000 AB/C for mHLA-DR; 336–1,126 CD4+ cells/μl).

We next evaluated circulating T lymphocyte functionality in patients at day 3 or 4 after ICU admission compared with controls through their capacity to produce effector cytokines and to proliferate following TCR stimulation.

**Figure 2:**
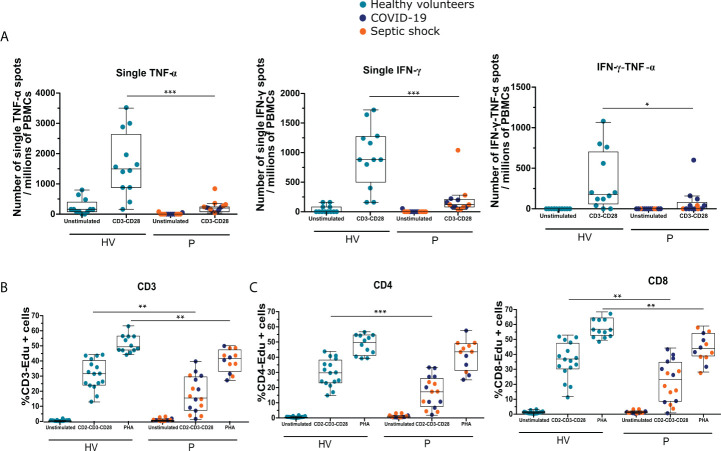
Patients with septic shock and COVID-19 in ICU are immunosuppressed and share T lymphocyte dysfunctions. The cytokine production and proliferation capacities of immune cells from patients (P) with septic shock and COVID-19 collected 3 or 4 days following ICU admission as well as and healthy volunteers (HVs) were analyzed using functional assays. **(A)** Numbers of single–TNF-α, single–IFN-γ, and double–IFN-γ–TNF-α spots per millions of PBMCs produced following overnight stimulation with anti-CD3 and anti-CD28 antibodies measured by ELISpot assay using PBMCs of patients with septic shock (n = 7) and COVID-19 (n = 6) or HVs (n =12). **(B)** Percentages of Edu+ CD3+ T cells following 3 days of PBMCs culture with anti–CD2-CD3-CD28 antibody–coated beads or phytohemagglutinin (PHA) at 4 μg/ml measured by flow cytometry in septic shock (n = 7) and COVID-19 (n = 9) patients or HVs (n = 16). **(C)** Percentages of Edu+ CD4+ or Edu+ CD8+ cells following 3 days of PBMCs culture with anti–CD2-CD3-CD28 antibody–coated beads (ratio 1:1) measured by flow cytometry in patients with septic shock (n = 7) and COVID-19 (n = 9) and HVs (n = 16). Data are represented as Tukey box plot. The non-parametric Mann–Whitney test was performed to compare HVs and patients. * p < 0.05, ** p < 0.01, and *** p < 0.001.

As shown in **Figure 2A**, when measuring the number of cells producing TNF-α and/or IFN-γ by ELISpot, we observed that the numbers of cells producing only TNF-α or only IFN-γ were significantly decreased in patients compared with HVs (for single TNF-α, a median of 200 [80–240] and 1,500 [880–2,650] and, for single IFN-γ, a median of 120 [80–211.1] and 880 [500–1,270] for patients and HVs, respectively, p < 0.001). The number of cells producing both IFN-γ and TNF-α was also significantly reduced in patients compared with HVs (0 [0–80] and 180 [60–710] for patients and HVs, respectively, p < 0.05).

These results were confirmed through intracellular cytokine staining in CD4+ T cells. Percentages of total IFN-γ–, IFN-γ–TNF-α–, and IFN-γ–TNF-α–IL-2–producing CD4+ T cells in patients were significantly decreased compared with HVs (12.5% [7.7–17.3] versus 22.8% [16.8–32.3], 3% [1.8–4.1] versus 4.3% [3.2–5.7], and 7.8% [3.3–13.1] versus 17.9% [11.0–20.3], for patients and HVs, respectively; p < 0.01), whereas the percentage of CD4+ T cells negative for these three cytokines was significantly increased after stimulation (**Figure S1A**). In contrast to CD4+ T cells, no significant difference was observed between patients and HVs for CD8+ T cells (**Figure S1B**).

Finally, we evaluated T-cell proliferative capacity following stimulation. As shown in **Figure 2B**, the percentage of proliferative Edu+ CD3+ T cells was significantly lower after TCR or PHA stimulation in patients compared with HVs (15.7% [7.5–29.5] versus 31.5% [24.1–40.3], and 41.4% [33.0–47.2] versus 49.2% [47.1–56.2] for patients and HVs, respectively; p < 0.01). As in the analysis of cytokine production, proliferation of both CD4+ and CD8+ T cells was analyzed (**Figure 2C**). The population of Edu+ CD4+ cells was significantly decreased after TCR stimulation in patients compared with HVs (17.3% [7.6–26.1] versus 29.8% [23.2–38.1] for patients and HVs, respectively; p < 0.01), whereas only a trend was observed after stimulation with PHA. For CD8+ cells, the proliferation capacity was significantly decreased after PHA and TCR stimulations (43.0% [38.1–53.0] versus 55.8% [51.6–63.2] in presence of PHA, 22.5% [8.2–3.9] versus 36.1% [29.5–46.5] in presence of TCR stimulation, for patients and HVs, respectively; p < 0.01).

Overall, these results show that functionality of T lymphocytes was altered after bacterial and viral sepsis in accordance with the development of injury-induced immunosuppression. Sepsis-induced T lymphocyte alterations were characterized by a decreased cytokine production (mainly of IFN-γ and/or TNF-α, mostly for CD4+ T cells) and a decreased proliferation capacity (for both CD4+ and CD8+ T cells).

### The MVA-secreted hIL-7-Fc initiates signaling pathway of IL-7 through the phosphorylation of STAT5

**Figure 3:**
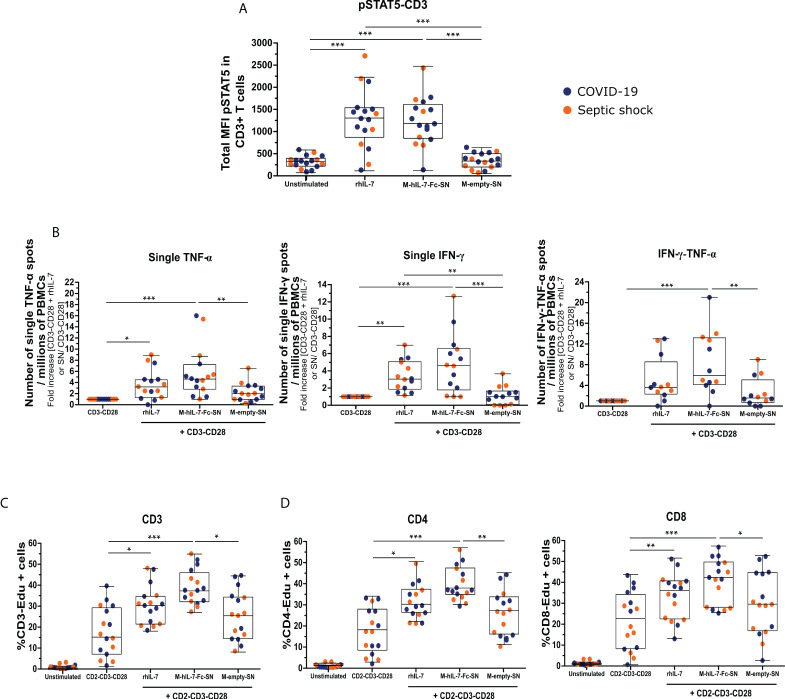
The MVA-secreted hIL-7-Fc restores *ex vivo* T lymphocyte function of patients with septic shock and COVID-19 at day 3 or 4 after ICU admission. **(A)** Total median of fluorescence (MFI) of pSTAT5 in CD3+ T cells measured by flow cytometry. Whole blood cells of patients with septic shock (n = 6) and COVID-19 (n = 11) were stimulated for 10 min with diluted supernatant from MVA-hIL-7-Fc (M-hIL-7-Fc-SN) and empty MVA (M-empty-SN) infection and rhIL-7 at 100 ng/ml as positive control. **(B)** Number of single–TNF-α, single–IFN-γ, and double–IFN-γ–TNF-α spots per millions of PBMCs. PBMCs of patients with septic shock (n = 7) and COVID-19 (n = 8) were stimulated overnight with anti-CD3 and anti-CD28 antibodies (basal condition of cytokine production) in addition to M-hIL-7-Fc-SN or M-empty-SN or rhIL-7 at 100 ng/ml. Number of spots were normalized using the basal condition. Percentage of Edu+ CD3+ T cells **(C)** and of Edu+ CD4+ or Edu+ CD8+ T cells **(D)**, measured by flow cytometry. PBMCs of patients with septic shock (n = 7) and COVID-19 (n = 9) were stimulated for 3 days using anti–CD2-CD3-CD28 antibody–coated beads (ratio 1:1) in addition to M-hIL-7-Fc-SN or M-empty-SN or rhIL-7 at 100 ng/ml. Data are represented as Tukey box plot. The non-parametric Friedman test followed by Dunn *post hoc* test for multiple comparisons was performed to compare all the conditions. * p < 0.05, ** p < 0.01, and *** p < 0.001.

The binding of IL-7 to its receptor initiates an intracellular signaling pathway through the phosphorylation of the STAT5 (pSTAT5). We investigated whether the hIL-7-Fc produced after MVA-hIL-7-Fc infection can initiate this pathway in primary cells from healthy donors and patients. pSTAT5 expression in CD3+ T cells was analyzed by flow cytometry. In all experiments, the commercially available rhIL-7 protein produced in *E.coli* was used as positive control. As expected, the rhIL-7 significantly increased the MFI of pSTAT5 in CD3+ T cells of HVs (**Figure S2A**) and patients (**Figure 3A**) compared with the unstimulated condition (MFI of 1,043 [753–1,353] versus 30.7 [12.7–75.7], p < 0.001, for HV, 1,297 [872–1m524] versus 333 [232–407], p < 0.001, for patients). Then, we assessed the effects of supernatants of cells infected with MVA on HVs and patients' cells. The MVA-secreted hIL-7-Fc increased pSTAT5 expression (769 [602–1,217]) in HVs compared with unstimulated condition, whereas the empty MVA supernatant had no effect (**Figure S2A**). Similar effects were observed when whole blood of patients was stimulated with the MVA supernatant containing hIL-7-Fc (MFI of 1,175 [848–1,600] versus 333 [232–407] for hIL-7-Fc supernatants and unstimulated condition, respectively, p < 0.001; **Figure 3A**). Thus, the hIL-7-Fc produced after MVA infection can efficiently initiate IL-7 intracellular signaling in T lymphocytes from patients with sepsis and controls.

### The MVA-secreted hIL-7-Fc enhances *ex vivo* T lymphocyte functions of patients with septic shock and critically ill COVID-19 at day 3 or 4 after ICU admission

The activity of cell supernatants containing the hIL-7-Fc was assessed on T lymphocytes from patients with septic shock and COVID-19. Cytokine production and T-cell proliferation after TCR stimulation were evaluated.

We confirmed that the rhIL-7 increased the numbers of single–TNF-α–, single–IFN-γ–, and double–IFN-γ–TNF-α–producing cells in patients (3.2-fold [1.3–4.5], p < 0.05; 3.0-fold [1.8–5.0], p < 0.01; and 3.6-fold [2.3–8.5], respectively) compared with TCR stimulation alone, as expected (**Figure 3B**). Then, supernatants from MVA infection were assessed on patients' cells. Compared with TCR stimulation alone, stimulation with the hIL-7-Fc supernatant led to a fold increase of 4.7 of the number of PBMCs, producing only TNF-α (p < 0.001), and to a 4.6-fold increase of single–IFN-γ spots (p < 0.001). Supernatant from empty MVA induced a minor production of single TNF-α, but a significant difference was observed in favor of the hIL-7-Fc supernatant (p < 0.01). No effect of supernatants from cells infected with the empty MVA on single–IFN-γ production was observed. The number of PBMCs producing both TNF-α and IFN-γ was significantly increased after MVA-secreted hIL-7-Fc stimulation (4.9-fold more than TCR stimulation alone, p < 0.001). The empty MVA supernatant induced a minor production of IFN-γ–TNF-α (1.5-fold more than TCR stimulation alone). Thus, for all parameters, the MVA-secreted hIL-7-Fc enhanced cytokine production in PBMCs from patients with viral and bacterial sepsis.

CD4+ and CD8+ T lymphocytes were analyzed by intracellular flow cytometry staining to determine if the MVA supernatant containing hIL-7-Fc could enhance cytokine production in both subpopulations (**Figures S3A–C**). No effect was observed on CD8+ T cells (data not shown). The hIL-7-Fc supernatant significantly increased the percentage of IFN-γ–TNF-α–IL-2–producing CD4+ cells, whereas only a trend was observed for IFN-γ– or IFN-γ–TNF-α–producing CD4+ cells.

Using Edu incorporation, the proliferation of T lymphocytes from patients was measured by flow cytometry (**Figure 3C**). As positive control, we confirmed that the rhIL-7 significantly increased the percentages of CD3, CD4, and CD8-Edu+ proliferating cells (30.6% [21.8–35.0], p < 0.05; 29.43% [25.5–36.5], p < 0.05; 34.3% [22.5–40.1], p < 0.01, respectively). The percentage of CD3-Edu+ proliferating cells increased significantly following MVA-secreted hIL-7-Fc stimulation compared with TCR stimulation alone (37.6% [32.4–46.1] versus 15.6% [7.5–29.5], respectively; p < 0.001). The empty MVA supernatant increased slightly the percentage of proliferating cells compared with TCR stimulation alone (25.8% [14.9–34.6], p < 0.05). The proliferation was significantly higher for the hIL-7-Fc supernatant compared with the empty MVA one. CD4+ and CD8 + T cells were analyzed to determine if the product could enhance proliferation capacities of both subpopulations (**Figure 3D**). The MVA supernatant containing hIL-7-Fc significantly increased the percentage of CD4-Edu+ or CD8-Edu+ compared with the TCR stimulation alone (37.0% [34.0–46.5] versus 17.3% [7.6–27.1] for CD4+ cells; 42.3% [27.9–49.2] versus 18.8% [8.4–32.7] for CD8+ cells; p < 0.001). A slight difference was observed after stimulation with empty MVA compared with TCR stimulation alone for both CD4+ and CD8+ T cells (26.4% [15.3–32.9] for CD4+ and 29.5% [17.1–44.2] for CD8+ cells) but a significant difference between the hIL-7-Fc and the empty MVA supernatant was observed (p < 0.01 for CD4+ cells and p < 0.05 for CD8+ cells).

Overall, these data indicate that hIL-7-Fc contained in the supernatant of MVA-hIL-7-Fc–infected cells can improve cytokine production and proliferation of T lymphocytes from both patients with septic shock and COVID-19.

### The MVA-secreted hIL-7-Fc enhances *ex vivo* lymphocytes functions of HVs

Interestingly, the secreted hIL-7-Fc was shown to also boost lymphocyte functions of healthy donors. IFN-γ and TNF-α productions were evaluated by ELISpot following the stimulation of PBMCs with hIL-7-Fc supernatant from MVA-infected cells (**Figure S2B**). A significant increase of the number of single–TNF-α–, single–IFN-γ–, and double–IFN-γ–TNF-α–producing cells was observed following the stimulation of PBMCs with hIL-7-Fc supernatant (increases of 4.7-fold, 6.2-fold, and 10.1-fold more, respectively, compared with TCR stimulation alone; p < 0.001). Increase of triple IFN-γ-TNF-α-IL-2 producing CD4+ cells was also observed by intracellular staining of cytokines (**Figure S2C**). In addition, the percentage of CD3+ Edu+, CD4+ Edu+, and CD8+ Edu+ cells was significantly increased following the MVA-secreted hIL-7-Fc stimulation compared with TCR stimulation alone (50.2% [47.9–52.7] versus 34.2% [26.1–41.8] for CD3+ T cells, 48.6% [47.6–52.0] versus 33.3% [24.6–39.5] for CD4+ cells, and 53.9% [52.1–58.8] versus 37.7% [33.0-48.4] for CD8+ cells; p < 0.001) (**Figures S2D, E**).

Overall, these data indicate that the hIL-7-Fc produced following MVA infection can enhance T lymphocyte function of healthy individuals illustrated by an improved capacity of cytokine production and cell proliferation.

## Discussion

The novel virotherapy MVA-hIL-7-Fc is being developed for the purpose of restoring immune-homeostasis in ICU patients with severe sepsis, who are in an established state of immunosuppression. In this bridging, proof-of-concept study, we assessed the capacity of the MVA-secreted hIL-7-Fc to restore *ex vivo* adaptive immune functions in two cohorts of immunosuppressed ICU patients: patients with septic shock with mainly a bacterial etiology (bacterial sepsis) and patients with COVID-19. The secreted hIL-7-Fc restored *ex vivo* immune defects of T lymphocytes in both cohorts as illustrated by an improved capacity of T cells to produce IFN-γ and/or TNF-α and improved proliferation capacity of both CD4+ and CD8+ T cells.

We have developed an innovative strategy to deliver in a broad range of organs the IL-7 cytokine with the goal to restore both innate and adaptive immunity of immunosuppressed patients with bacterial sepsis and COVID-19. Our virotherapy is based on the non-propagative MVA platform (31). Safety of the MVA vector has been well established in both healthy individuals and chronically infected patients (38, 39) [e.g., HIV-infected patients (40)]

Here, we assessed the immune-stimulating activity of the MVA-secreted hIL-7-Fc *ex vivo* on T lymphocytes of immunosuppressed patients with septic shock and COVID-19. First, we evaluated the capacity of the MVA-secreted hIL-7-Fc to trigger the IL-7 signaling pathway evidenced by an increased expression of the phosphorylated STAT5 in T lymphocytes (41). The MVA-secreted hIL-7-Fc initiated the signaling pathway of the IL-7 by inducing the expression of the pSTAT5 in CD3+ T lymphocytes in both ICU patients with septic shock and COVID-19. In patients with septic shock, Demaret et al. (42) reported an increase expression of the phosphorylated STAT5 in CD4+ effector cells of patients with sepsis following stimulation with a rhIL-7. Second, we investigated the capacity of the MVA-secreted hIL-7-Fc to boost T lymphocyte functions of the two sepsis cohorts. Decreased IFN-γ production by T lymphocytes is a hallmark of exhausted T lymphocytes (10, 16, 43). Our data showed that the hIL-7-Fc–containing supernatant harbored the capacity to restore the production of IFN-γ and/or TNF-α cytokines by T lymphocytes from both patients with septic shock and COVID-19. Our observation is concordant with the enhanced IFN-γ production by lymphocytes reported in *ex vivo* studies using rhIL-7 for immunosuppressed patients with septic shock (18, 44, 45) and, more recently, also for patients with critically ill COVID-19 (17, 19). IFN-γ is an important cytokine, and its *ex vivo* production by T lymphocytes has been shown to be reduced in sepsis (16). IFN-γ plays a role in the activation of monocytes and increases their antigen-presentation and phagocytic capacities (46). As such, IFN-γ is proposed as an immunoadjuvant therapy in sepsis (12). Recently, in case series, multiple injections of IFN-γ were shown to increase mHLA-DR expression and improve clinical status of most treated immunosuppressed patients with sepsis (47). In two case series including critically ill COVID-19, a rapid decline in SARS-CoV-2 viral load and/or clinical improvement were observed following multiple injection of IFN-γ (48, 49). Thus, increased production of IFN-γ following stimulation with the MVA-secreted hIL-7-Fc is an asset. The increase of lymphocyte-driven production of TNF-α observed with the hIL-7-Fc–containing supernatant is original. In recent study in patients with COVID-19 and sepsis, *ex vivo* stimulation with lipopolysaccharides to trigger TNF-α production by monocytes in presence of rhIL-7 failed to improve the cytokine production (7, 44). TNF-α is an important cytokine stimulating APCs to produce pro-inflammatory cytokine and promoting immune responses against pathogens (50). In addition, the MVA-secreted hIL-7-Fc was found to significantly improve the proliferation of both CD4+ and CD8+ T cells, which is another aspect of injury-induced immunosuppression. Although it is known that *ex vivo* stimulation with rhIL-7 can enhance the proliferative capacity of T lymphocyte from sepsis patients (18), it has only been recently documented for patients with COVID-19 (18, 19). However, T-cell responses were only assessed using TCR stimulation. It might be interesting to assess antigen-specific T-cell stimulation. It would illustrate that specific T responses could be restored by hIL-7-Fc as well. Overall, the present results confirmed that a MVA-driven virotherapy expressing an hIL-7-Fc is effective at enhancing T lymphocyte–mediated adaptive immunity in both ICU patients with septic shock and COVID-19. These results are aligned with our previous observations made in septic mice in which a single injection of the MVA-hIL-7-Fc enhanced the polyfunctionality of T lymphocytes especially illustrated by a significant increased number of IFN-γ–producing cells (32).

Interestingly, Fend et al. (37) demonstrated that, following the IV injection of an MVA-luciferase in mice, the MVA was mostly detected in the lung, the liver, and the spleen of animals. Here, we transduced primary cells and cell lines from hepatic and monocytic origin with the MVA-hIL-7-Fc to mimic an IV injection *in vivo* and characterized the produced and secreted hIL-7-Fc. Our data showed that the detected hIL-7-Fc in all four cells supernatant displayed a similar feature, i.e., was dimeric, glycosylated, and biologically active. These data are concordant with previous observations made following infection of a pneumocyte human cell line and primary murine hepatocytes with the MVA-hIL-7-Fc (32). Taken together, these data indicate that an IV injection of the MVA-hIL-7-Fc, expected to result in infection of a variety of organs and cells, will deliver a homogeneous and broadly functional hIL-7-Fc.

In our study, we assessed the activity of the MVA-secreted hIL-7-Fc on T lymphocytes of ICU sepsis patients infected or not with SARS-CoV-2. It was important to verify that T lymphocytes were dysfunctional in both cohorts. We confirmed the profound lymphopenia affecting the two cohorts with less than 500 CD4+ cells/μl as previously described (8, 51–53). In addition, we observed a significant decreased of IFN-γ– and/or TNF-α–producing cells in both types of patients. Our data are concordant with recent studies describing a reduced capacity of IFN-γ and/or TNF-α production by T lymphocytes in similar patients (7, 17, 19, 44). As we observed in our study, these studies also reported an altered proliferation of both CD4+ and CD8+ T cells in both cohorts of patients (18, 19, 54).

### Limitations

This study presents some limitations. The observed effects should be consolidated in a larger number of patients. Our study focused on the hIL-7-Fc produced in supernatants of infected primary hepatocytes. It should be expanded to the evaluation of supernatants obtained from monocytes infected by the MVA-hIL-7-Fc. In addition, as patients received different treatments during their ICU stay (e.g., corticosteroids and antibiotics) depending on sepsis origin, putative impact of treatments on hIL-7-Fc effect could be assessed in further studies. In addition, hIL-7-Fc effect on T-cell apoptosis is another aspect that would deserve additional exploration (41). Last, because hIL-7-Fc supernatants were only assessed on T-cell sampled during first days in ICU, it will be of utmost importance in further study to evaluate the capacity of the hIL-7-Fc to boost T lymphocyte function in convalescent patients.

## Conclusions

This bridging study aimed at assessing the capacity of a novel MVA-based IL-7 expressing virotherapy to restore adaptive immunity of immunosuppressed ICU patients with septic shock and COVID-19. Our results show that the hIL-7-Fc produced by the MVA-hIL-7-Fc initiates the IL-7 signaling pathway through the phosphorylation of STAT5 and restored *ex vivo* T lymphocyte functions of these patients. These data are supportive of the clinical development of the MVA-hIL-7-Fc for the benefit of ICU patients with septic shock and COVID-19.

## RICO study group

The names of the individual members of the RICO study group as listed below should be searchable through their individual PubMed records:

- Hospices Civils de Lyon, Lyon-Sud University Hospital, Immunology Laboratory: Remi Pescarmona, Lorna Garnier, Christine Lombard, Magali Perret, and Marine Villard
- Joint Research Unit HCL-bioMérieux: Valérie Cheynet and Filippo Conti
- Centre d'Investigation Clinique de Lyon (CIC 1407 Inserm): Marie Groussaud, Marielle Buisson, Laetitia Itah, and Inesse Boussaha
- Hospices Civils de Lyon, Edouard Herriot Hospital, Immunology Laboratory: Françoise Poitevin-Later, Christophe Malcus, and Morgane Gossez
- RICO clinical investigators: Florent Wallet, Marie-Charlotte Delignette, and Frederic Dailler
- Hospices Civils de Lyon, Edouard Herriot Hospital, Medical intensive Care Department: Marie Simon, Auguste Dargent, Pierre-Jean Bertrand, Neven Stevic, and Marion Provent
- Hospices Civils de Lyon, Edouard Herriot Hospital, Anesthesia and Critical Care Medicine Department: Laurie Bignet and Valérie Cerro
- Hospices Civils de Lyon, Croix-Rousse University Hospital, Medical intensive Care Department: Jean-Christophe Richard, Laurent Bitker, Mehdi Mezidi, and Loredana Baboi

## Data availability statement

The raw data supporting the conclusions of this article will be made available by the authors, without undue reservation.

## Ethics statement

This project was reviewed and approved by Institutional Review Board for ethics (“Comité de Protection des Personnes Ouest II”, n° RCB: 2019-A00210-57 and “Comité de Protection des Personnes Ile de France 1”, n° RCB: 115 2020-A01079-30). Written informed consent for participation was not required for this study in accordance with the national legislation and the institutional requirements.

## Author contributions

MC, GM, FC, PM, GI, and FV designed the study. MC performed experiments and data collection and analysis. J-BM designed the biochemical study. A-CL performed patients' inclusion and sample and clinical data collection. The first draft was written by MC, and FC, GM, GI, and FV contributed to other versions of the manuscript. All authors read, reviewed, and approved the final manuscript.

## Funding

The project was partially granted by ANRT (Grant no. 2019/0650- PhD training of Morgane Crausaz). This study was supported by funds from Hospices civils de Lyon and Transgene.

## Acknowledgments

The authors would like to thank the Vectorology lab of Transgene for the MVA production and characterization. We thank Patricia Kleinpeter, Charles-Antoine Coupet, and Alexeï Evlachev for technical support. The authors would like to thank the clinical teams from all ICUs in HCL for inclusion of patients and the technical staff from Immunology lab of E. Herriot hospital for the routine immunological data analysis.

## Conflict of interest

MC, J-BM, PM, and GI were employees of Transgene SA when the work was performed. Transgene SA is a publicly traded French biopharmaceutical company, with Institut Merieux as the major shareholder. MC, GM, FC and A-CL work at EA7426, a joint unit including University, Hospital and bioMérieux, but are not employed by bioMérieux.

The remaining authors declare that the research was conducted in the absence of any commercial or financial relationships that could be construed as a potential conflict of interest.

The authors declare that this study received f​​​​unding from Transgene SA. The funder had the following involvement with the study: collection of data, analysis, interpretation of data and writing of the article.

## Publisher’s note

All claims expressed in this article are solely those of the authors and do not necessarily represent those of their affiliated organizations, or those of the publisher, the editors and the reviewers. Any product that may be evaluated in this article, or claim that may be made by its manufacturer, is not guaranteed or endorsed by the publisher.
