# Supplementary material for: A novel virotherapy encoding human interleukin-7 improves ex vivo T lymphocyte functions in immunosuppressed patients with septic shock and critically ill COVID-19
Source: Front Immunol. 2022 Aug 15;13:939899. doi: 10.3389/fimmu.2022.939899 (PMC9422896; doi:10.3389/fimmu.2022.939899)
Supplement: Supplementary file 1 [file Image_1.pdf]

SUPPLEMENTARY FIGURES 1

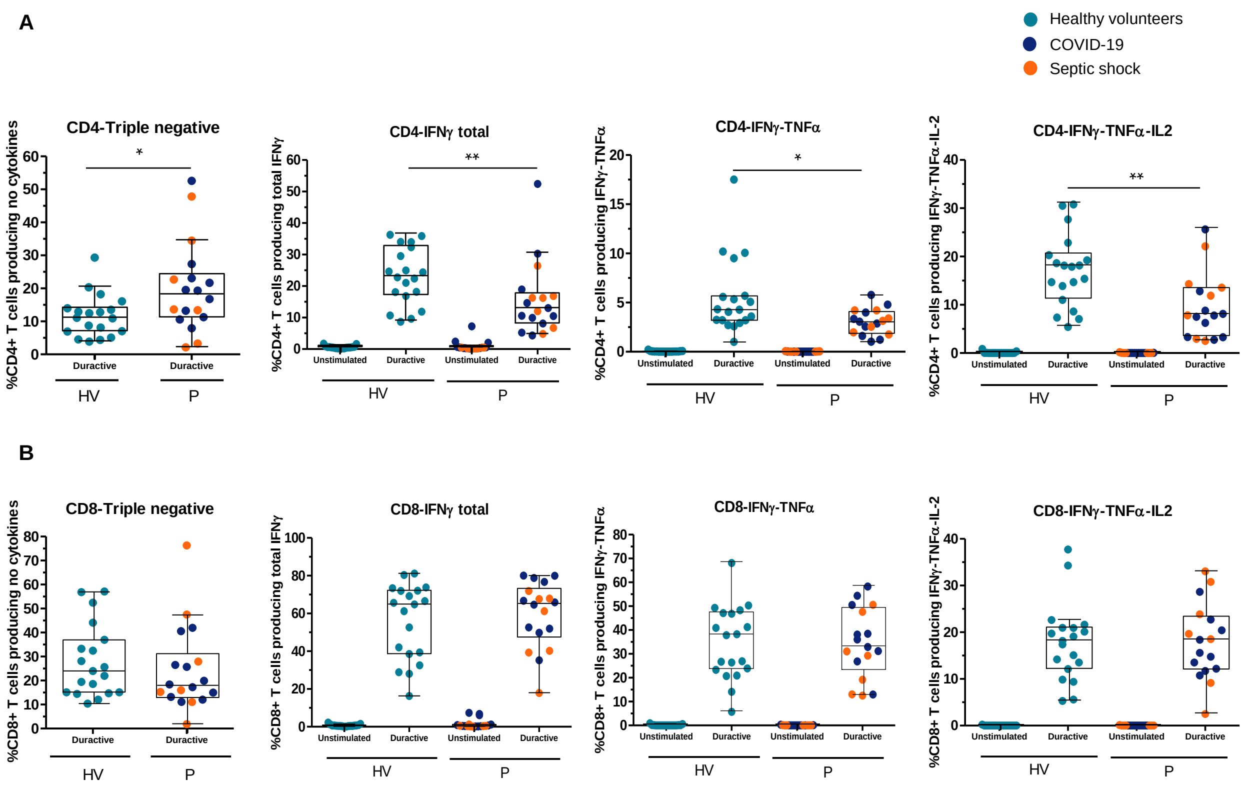

Supplementary figure 1. Septic shock and COVID-19 patients in ICU display lymphocyte dysfunctions with a decreased production of cytokines by CD4+ T cells. Whole blood cells of septic shock (n=7) and COVID-19 (n=10) patients or HV (n=17) were stimulated 3 hours with the Duractive 1 stimulating agent (PMA-Ionomycin). Percentage of triple negative, total IFN-γ, double IFN-γ TNF-α and triple IFN-γ TNF-α IL-2 producing CD4+ **(a)** or CD8+ **(b)** T cells after PMA-Ionomycin stimulation measured by intracellular staining of cytokines. Data are represented as Tukey box plots. The nonparametric Mann Whitney test was performed to compare HV and patients.

SUPPLEMENTARY FIGURES 2

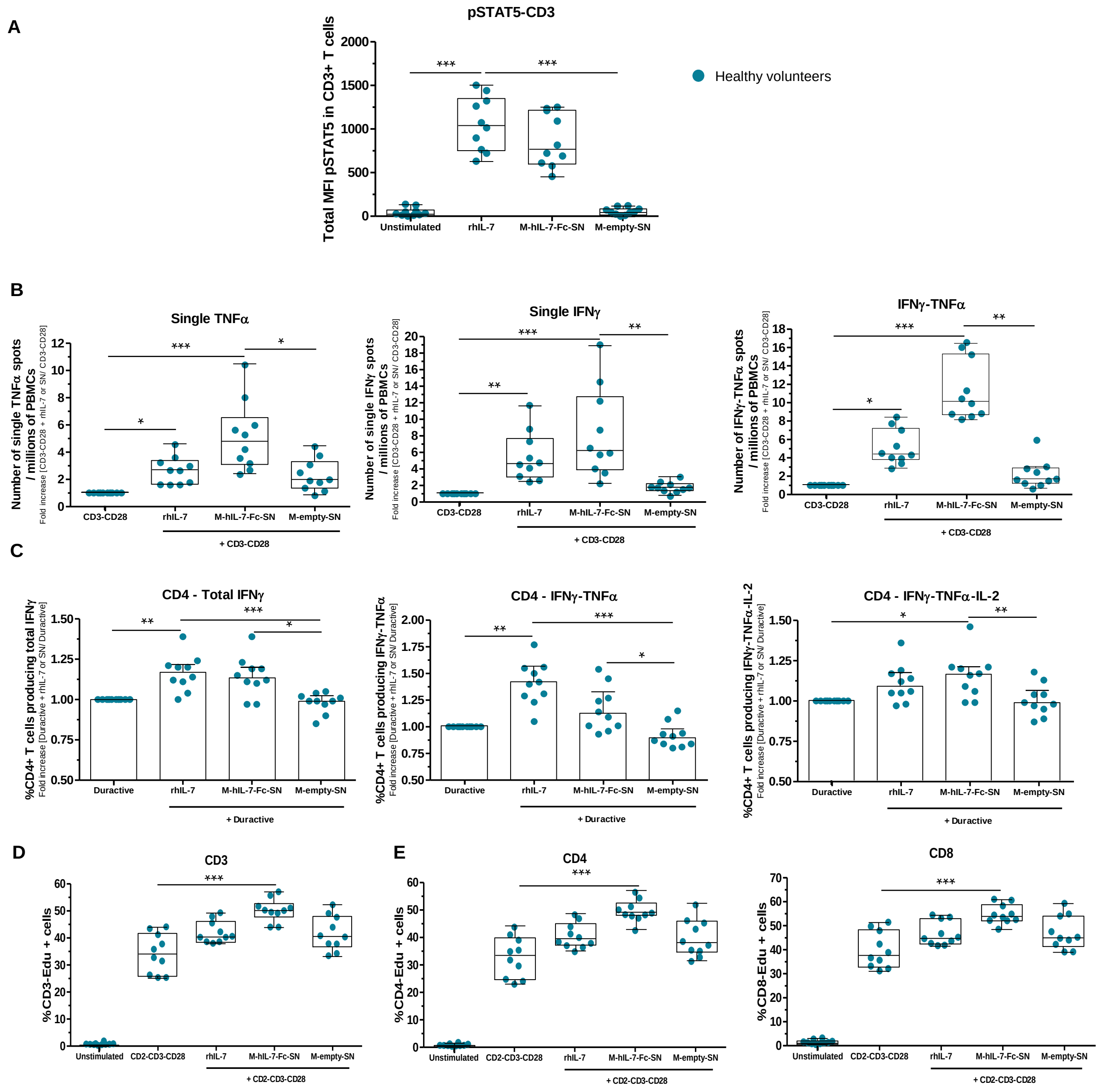

SUPPLEMENTARY FIGURES 2

Supplementary figure 2. The MVA-secreted hIL-7-Fc enhances *ex vivo* T lymphocyte function of healthy volunteers. **(a)** Total median of fluorescence (MFI) of pSTAT5 in CD3+ T cells measured by flow cytometry. Whole blood cells of HV (n=10) were stimulated 10 minutes with diluted MVA-hIL-7-Fc supernatant (M-hIL-7-Fc-SN) or empty MVA supernatant (M-empty-SN) or rhIL-7 at 100 ng/mL **(b)** Number of single TNF-α, single IFN-γ and double IFN-γ TNF-α spots per millions of PBMCs. PBMCs of HV (n=10) were stimulated overnight with anti-CD3 and anti-CD28 antibodies (basal condition of cytokine production) in addition to M-hIL-7-Fc-SN or M-empty-SN or rhIL-7 at 100 ng/mL. Number of spots of the different treatments were normalized using the basal condition. **(c)** Percentage of total IFN-γ, double IFN-γ TNF-α and triple IFN-γ TNF-α IL-2 CD4+ - producing cells measured by flow cytometry. Whole blood cells of HV (n=10) were stimulated with PMA-Ionomycin (Duractive 1, basal condition of cytokine production) in addition to M-hIL-7-Fc-SN or M-empty-SN or rhIL-7 at 100 ng/mL. Percentages were normalized using the basal condition. **(d)** Percentage of Edu+ CD3+ T cells and **(e)** of Edu+ CD4+ and Edu+ CD8+ T cells measured by flow cytometry. PBMCs of HV (n=10) were stimulated 3 days using anti-CD2-CD3-CD28 antibody coated beads (ratio 1:1) in addition to M-hIL-7-Fc-SN or M-empty-SN or rhIL-7 at 100 ng/mL. Data are represented as Tukey box plots or bar plots representing the median + the interquartile range. The nonparametric Friedman test was performed followed by Dunn post-hoc pairwise multiple comparison to compare all the conditions. * $p < 0.05$, ** $p < 0.01$ and *** $p < 0.001$.

SUPPLEMENTARY FIGURES 3

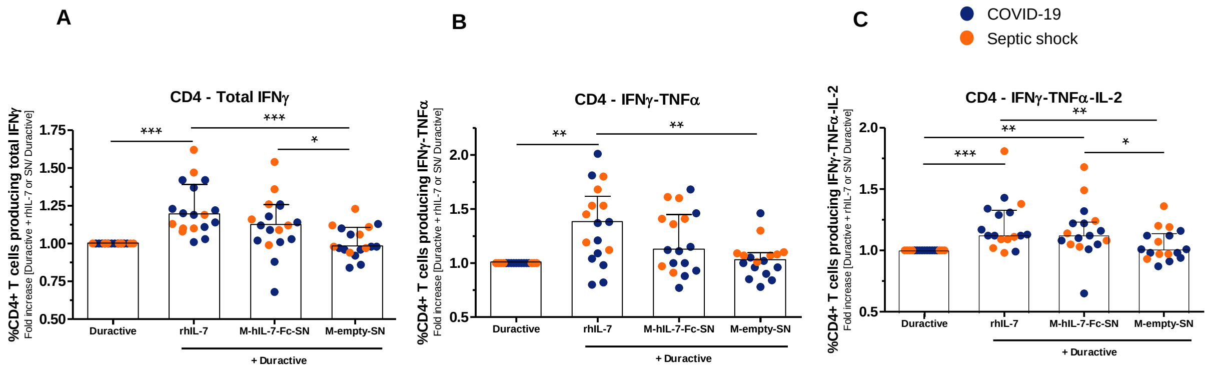

Supplementary figure 3. The MVA-secreted hIL-7-Fc increased cytokine production by CD4+ T cells of septic shock and COVID-19 patients. **(a)** Percentage of total IFN-γ, **(b)** double IFN-γ TNF-α and **(c)** triple IFN-γ TNF-α IL-2 producing-CD4+ T cells measured by flow cytometry. Whole blood cells of septic shock (n= 7) and COVID-19 (n=10) patients were stimulated 3 hours with PMA-Ionomycin (Duractive 1, basal condition of cytokine production) in addition to M-hIL-7-Fc-SN or M-empty-SN or rhIL-7 at 100 ng/mL. Percentages were normalized using the basal condition. Data are represented as bar plots representing the median + the interquartile range. The nonparametric Friedman test was performed followed by Dunn post-hoc pairwise multiple comparison to compare all the conditions. * $p < 0.05$, ** $p < 0.01$ and *** $p < 0.001$.
